# A biphasic modeling framework for arterial compressibility under steady axisymmetric deformation

**DOI:** 10.1007/s10237-026-02082-6

**Published:** 2026-06-05

**Authors:** Takeo Fujiwara, Shukei Sugita, Shigeo Wada, Tomohiro Otani

**Affiliations:** 1 Department of Mechanical Science and Bioengineering, Graduate School of Engineering Science, The University of Osaka, 1-3, Machikaneyamacho, Toyonaka, 560-8531 Osaka Japan; 2 Department of Electrical and Mechanical Engineering, Graduate School of Engineering, Nagoya Institute of Technology, Gokiso-cho, Showa-ku, Nagoya, 466-8555 Aichi Japan

**Keywords:** Artery, Compressibility, Biphasic model, Interstitial fluid, Pressure-inflation test

## Abstract

Arterial walls contain large amounts of water and have conventionally been modeled as incompressible. However, recent experimental studies have reported non-negligible arterial compressibility, with volumetric changes on the order of 10% or larger depending on loading conditions. To clarify the mechanical origin and its implications, this study develops a biphasic modeling framework for arterial mechanics, in which apparent compressibility arises from interstitial fluid transfer within the arterial wall. The arterial wall is modeled as a saturated biphasic material consisting of a solid skeleton and interstitial fluid, in which the solid skeleton is modeled as an anisotropic, hyperelastic material with macroscopic volumetric deformability, and the fluid motion is governed by Darcy’s law. Assuming steady, axisymmetric plane-strain deformation, the resulting nonlinear mechanical equilibrium is reduced to a one-dimensional radial boundary-value problem and solved numerically using a finite element method. Systematic parametric analyses demonstrate that radial and circumferential deformations, as well as the resulting volumetric changes, are consistent with experimentally observed mean values, with deviations within 2% under the same loading conditions. Such volumetric expansion, driven by the hydrostatic pressure of the interstitial fluid, induces tensile stress components in the radial direction within the solid skeleton, revealing a mechanical consequence of fluid–solid interactions that is not directly accessible from apparent deformation measures alone. These findings suggest that biphasic modeling provides a mechanically interpretable framework for examining arterial wall responses in regimes where fluid–solid interactions are relevant.

**Supplementary Information:**

The online version contains supplementary material available at 10.1007/s10237-026-02082-6.

## Introduction

The arterial wall contains substantial amounts of intracellular and extracellular water (70–80% by wet weight (Humphrey 2002)), and interstitial fluid movement plays an essential role in arterial physiology (Harrison and Massaro 1976; Kenyon 1979). Despite this, most constitutive models have conventionally treated the arterial wall as incompressible or nearly incompressible (Fung 1993; Holzapfel et al. 2000; Holzapfel and Ogden 2010a). This is based on earlier experimental evidence (Carew et al. 1968; Chuong and Fung 1984) reporting that the volumetric change of the arterial wall was secondary to mechanical deformation, as extensively reviewed in Humphrey (2002).

However, several experimental studies have reported non-negligible compressibility of arterial walls (Di Puccio et al. 2012; Yosibash et al. 2014; Skacel and Bursa 2016; Nolan and McGarry 2016; Yossef et al. 2017; Sugita et al. 2020; Skacel and Bursa 2022). Yosibash et al. (2014) critically reviewed earlier in vitro experimental data regarding arterial compressibility, and further reported, based on their own pressure-inflation experiments, that arterial volume changes of approximately 2–6% can occur during inflation within the physiological transmural pressure range of 50–200 mmHg. Importantly, they also pointed out that significant volumetric changes arose at lower transmural pressures in their subsequent study (Yossef et al. 2017), although quantitative data in this regime were not shown. To address arterial deformation under low-pressure conditions, we conducted in vitro inflation experiments (Sugita et al. 2020) using an experimental setup specifically designed for quantitative evaluation under a wide range of transmural pressures in Sugita and Matsumoto (2017). The results demonstrated that arterial volume increased by nearly 40% as transmural pressure rose from 15 to 80 mmHg (Sugita et al. 2020). These findings are also consistent with compression tests reported by Nolan and McGarry (2016), which reported volumetric changes on the order of 10% under compressive stresses below 10 kPa (75 mmHg). Together, these experimental observations indicate that arterial walls exhibit pronounced and nonlinear compressibility, particularly in the low-pressure regime.

To describe the mechanical interaction between solid tissues and interstitial fluid that underlies such compressible behavior, biphasic (poroelastic) modeling provides a natural theoretical framework (Boer 2000; Ehlers and Bluhm 2002; MacMinn et al. 2016). In this framework, a deformable solid skeleton is saturated with interstitial fluid, allowing coupled solid deformation and fluid transport to be considered within a unified mechanical description. This theoretical framework has also been extensively developed for biological soft tissues, providing a general basis for fluid-saturated deformable materials (Mow et al. 1984; Simon et al. 1993; Levenston et al. 1998; Ehlers and Markert 2001; Ehlers et al. 2009; Karajan 2009; Ateshian 2009; Tully and Ventikos 2011). In arterial biomechanics, however, biphasic modeling has been predominantly employed to investigate mass transport phenomena within the arterial wall, such as interstitial fluid flow or solute exchange (Altundemir et al. 2024; Kailash et al. 2025a, b; Chooi et al. 2016; Badia et al. 2009), and thus its mechanical implications for arterial deformation have received comparatively less attention. As a result, even within biphasic formulations, the solid skeleton has often been treated as nearly incompressible, which may underestimate the experimentally observed arterial compressibility and make its underlying mechanical implications unclear.

In this study, we develop a biphasic modeling framework to interpret experimentally observed arterial compressibility and to examine its mechanical implications. Based on the experiments in Sugita et al. (2020), arterial deformation during inflation is assumed to be steady, axisymmetric, and in a plane-strain state. Under these assumptions, the governing equations of biphasic materials can be reduced to a one-dimensional form in the radial direction, even under finite-deformation theory. This reduction enables systematic analyses of material parameter sensitivity and provides mechanical insights into the effects of biphasic interactions on arterial deformation.

Within this context, Auton and MacMinn (2017) derived a general theoretical framework for axisymmetric biphasic materials under finite deformation using an Eulerian formulation. Their framework established a rigorous theoretical foundation for steady-state poroelastic deformation and clarified the fundamental structure of the governing equations. However, its extension to arterial mechanics poses practical challenges, particularly when incorporating complex constitutive features such as anisotropic hyperelasticity and intrinsic residual stress. These complexities make the derivation of analytical or semi-analytical solutions difficult and limit systematic parametric investigations. To address this issue, we build upon this theoretical framework and reformulate the problem within a finite element setting. This reformulation enables robust nonlinear solutions and systematic parametric analyses, while retaining the essential structure of the original biphasic theory.

The remainder of this paper is organized as follows. Section 2 presents a problem statement, theoretical and computational formulations, and procedures to analyze the sensitivities of material parameters. The computational results are presented in Sect. 3, and the discussion and concluding remarks are provided in Sects. 4 and 5, respectively.

## Methods

### Problem statement

**Fig. 1:**
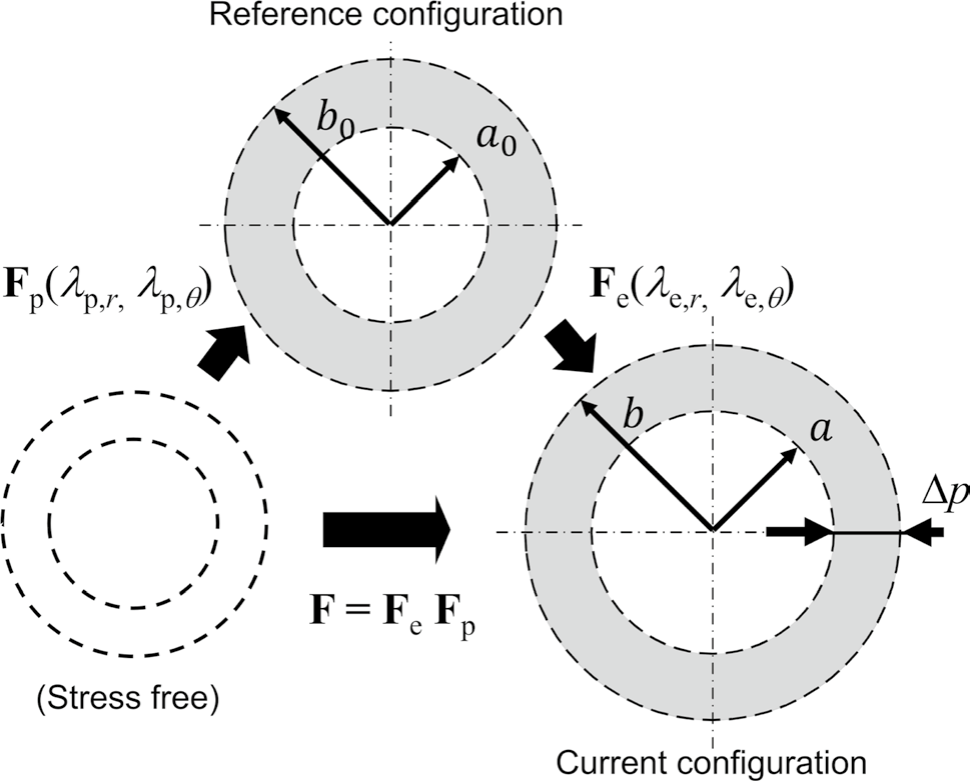
Schematic of the axisymmetric arterial model. The arterial wall is represented as a straight cylindrical tube under plane-strain conditions. The arterial wall with no transmural pressure is taken as a reference configuration and its deformation is described by the multiplicative decomposition of the deformation gradient tensor $\textbf{F}=\textbf{F}_\text {e}\textbf{F}_\text {p}$

The arterial geometry is represented as an axisymmetric straight cylinder with inner and outer radii $(a_0,b_0)$ in the reference configuration and (a, b) in the current configuration, respectively (Fig. 1). A cylindrical coordinate system (*o*–$r\theta z$) is introduced, where the *z*-axis coincides with the centerline of the artery. An arterial segment is subjected to a transmural pressure difference $\Delta p$ ($\Delta p=0$ in the reference configuration and $\Delta p>0$ acting in the outward direction), consistent with in vitro pressure–inflation experiments in Sugita et al. (2020). Under these conditions, the deformation is assumed to be steady, axisymmetric, and in a plane-strain state along the axial direction.

The arterial tissue is modeled as a fully saturated two-phase poroelastic material consisting of a porous solid skeleton and interstitial fluid. Both the solid and fluid constituents (phases) are assumed to be intrinsically incompressible. In this framework, the porous solid skeleton can undergo macroscopic volumetric deformation through changes in porosity (i.e., in the volume fractions of the solid and fluid phases). The volume fractions of the fluid and solid skeleton are denoted by $(\phi _{\text {f},0},\phi _{\text {s},0})$ in the reference configuration and $(\phi _\text {f},\phi _\text {s})$ in the current configuration, such that

1 $$\begin{aligned} \phi _{\text {s},0}+\phi _{\text {f},0}=1,\,\phi _{\text {s}}+\phi _{\text {f}}=1. \end{aligned}$$

In the following formulation, the kinematics and governing equations are expressed using an Eulerian description following the framework of Auton and MacMinn (2017). Within this description, the primary kinematic variables are the radial displacement of the solid skeleton $u_\text {s}$ and the radial velocity of the interstitial fluid $v_\text {f}$. This choice allows a straightforward representation of the coupled mechanics of solid–fluid mixtures (MacMinn et al. 2016). In this framework, $u_\text {s}$ at position $r\in [a,b]$ is defined as

2 $$\begin{aligned} u_\text {s}(r) = r - R(r), \end{aligned}$$

where *R*(*r*) denotes the reference position of the material that is located at position *r* in the current configuration, and $R(r)=r$ in the reference configuration (Auton and MacMinn 2017). Additionally, anisotropic hyperelastic properties of the solid skeleton, as well as the pre-stretch in the reference configuration to describe residual stresses, are incorporated in the formulation.

### Formulation

#### Kinematics

The kinematics of the solid skeleton with pre-stretch are described using a multiplicative decomposition of the deformation gradient tensor $\textbf{F}$ as $\textbf{F}=\textbf{F}_\text {e}\textbf{F}_\text {p}$, where $\textbf{F}_\text {e}$ represents the elastic deformation from the reference configuration and $\textbf{F}_\text {p}$ denotes the initial pre-stretch in the reference configuration. Under axisymmetric deformation, these components are written as

3 $$\begin{aligned} \textbf{F}_\text {e}&=\text {diag}(\lambda _{\text {e},r},\lambda _{\text {e},\theta },\lambda _{\text {e},z}), \end{aligned}$$

4 $$\begin{aligned} \textbf{F}_\text {p}&=\text {diag}(\lambda _{\text {p},r},\lambda _{\text {p},\theta },\lambda _{\text {p},z}), \end{aligned}$$

where $\lambda _{i,r}$, $\lambda _{i,\theta }$, and $\lambda _{i,z}$ ($i=\text {e,p}$) are the radial, circumferential, and axial stretch ratios associated with elastic deformation and pre-stretch, respectively. Under the plane-strain deformation assumption ($\lambda _{\text {e},z}=\lambda _{\text {p},z}=1$), the elastic stretch ratios in the Eulerian description are given by

5 $$\begin{aligned} \lambda _{\text {e},r} = \left( 1-\frac{\partial u_\text {s}}{\partial r}\right) ^{-1}, \lambda _{\text {e},\theta } = \left( 1-\frac{u_\text {s}}{r}\right) ^{-1}. \end{aligned}$$

Using these definitions, the total stretch ratios $\lambda _r$ and $\lambda _\theta$ are expressed as

6 $$\begin{aligned} \lambda _r = \lambda _{\text {e},r}\lambda _{\text {p},r}, \lambda _\theta =\lambda _{\text {e},\theta }\lambda _{\text {p},\theta }, \end{aligned}$$

and the volume change ratio $J = \text {det}(\textbf{F})$ is given by

7 $$\begin{aligned} J = \lambda _r\lambda _\theta . \end{aligned}$$

Next, the kinematics of the interstitial fluid are described by steady radial flow, which is represented as a line source with flow rate per unit length *Q* under the axisymmetric assumption. From mass conservation, the radial velocity of the interstitial fluid $v_\text {f}$ satisfies

8 $$\begin{aligned} \phi _\text {f}v_\text {f} = \frac{Q}{2\pi r}. \end{aligned}$$

Here, *Q* denotes an effective interstitial fluid flow rate per unit axial length in the axisymmetric setting. It represents the net radial fluid transport across the arterial wall thickness under steady pressurization, such as transmural filtration and fluid exchange within and around the arterial wall.

The volume fractions of the fluid and solid phases, $\phi _\text {f}$ and $\phi _\text {s}$, are determined from the solid displacement $u_\text {s}$. The volume change ratio from the reference configuration, $J_\text {e}$, is defined as

9 $$\begin{aligned} J_\text {e}:= \frac{V}{V_0} = \frac{V_\text {s}/\phi _\text {s}}{V_{\text {s},0}/\phi _{\text {s},0}} =\frac{\phi _{\text {s},0}}{\phi _\text {s}}= \frac{1-\phi _{\text {f},0}}{1-\phi _\text {f}}, \end{aligned}$$

where *V* and $V_0$ denote the current and reference volumes of the solid–fluid mixture, and $V_\text {s}$ and $V_{\text {s},0}$ are the current and reference volumes of the solid phase, respectively. Owing to the incompressibility of the solid phase, $V_\text {s}=V_{\text {s},0}$ holds. Using $J_\text {e}=\lambda _{\text {e},r}\lambda _{\text {e},\theta }$ together with eq. 5 in the Eulerian description, the fluid volume fraction $\phi _\text {f}$ is expressed in terms of $u_\text {s}$ as

10 $$\begin{aligned} \frac{\phi _\text {f}-\phi _{\text {f},0}}{1-\phi _{\text {f},0}}=\frac{1}{r}\frac{\partial }{\partial r}\left( ru_\text {s}-\frac{1}{2}u_\text {s}^2\right) . \end{aligned}$$

#### Momentum balance

The quasi-static momentum balance of the solid–fluid mixture is expressed as

11 $$\begin{aligned} \nabla \cdot \boldsymbol{\sigma } = \textbf{0}, \end{aligned}$$

where $\boldsymbol{\sigma }$ denotes the Cauchy stress (total stress) tensor of the mixture. The total stress is decomposed into Terzaghi’s effective stress of the solid skeleton ${\sigma }^{\prime }$ and the pore fluid pressure *p* as

12 $$\boldsymbol{\sigma }=\boldsymbol{\sigma }^{\prime }-p\textbf{I}.$$

Under axisymmetric conditions, eq. 11 reduces in cylindrical coordinates to

13 $$ \frac{\partial \sigma ^{\prime }_r}{\partial r}+\frac{\sigma ^{\prime }_r-\sigma ^{\prime }_\theta }{r}-\frac{\partial p}{\partial r} = 0,$$

where $\sigma ^{\prime }_r$ and $\sigma^{\prime }_\theta$ are the radial and circumferential components of $\boldsymbol{\sigma }^{\prime }$.

The radial pressure gradient is related to interstitial fluid flow through Darcy’s law, given by

14 $$\begin{aligned} \phi _\text {f} v_\text {f} = -k\frac{\partial p}{\partial r}, \end{aligned}$$

where *k* denotes the hydraulic permeability of the porous solid skeleton. Substituting eqs. 8 and 14 into eq. 13, the pressure gradient term can be eliminated, yielding

15 $$\frac{\partial \sigma^{\prime }_r}{\partial r}+\frac{\sigma^{\prime }_r-\sigma^{\prime }_\theta }{r}+\frac{1}{k}\frac{Q}{2\pi r} = 0.$$

#### Constitutive law: solid skeleton

The solid skeleton of the artery is modeled as an anisotropic hyperelastic material to describe the macroscopic mechanical behavior of arterial tissue. The strain energy density function *W* is decomposed into deviatoric and volumetric parts, $W=W_\text {dev}+W_\text {vol}$. The deviatoric part $W_\text {dev}$ is described using a Fung-type anisotropic model (Chagnon et al. 2015), given by

16 $$\begin{aligned} W_\text {dev} = \frac{C}{2}(\exp {(\tilde{Q}_W)}-1), \end{aligned}$$

where *C* is a material parameter and $\tilde{Q}_W$ is defined following (Chuong and Fung 1984) as

17 $$\begin{aligned} \begin{aligned} \tilde{Q}_W =&b_1\tilde{E}^2_\theta + b_2\tilde{E}^2_z + b_3\tilde{E}^2_r + 2b_4\tilde{E}_\theta \tilde{E}_z \\&+ 2b_5\tilde{E}_r\tilde{E}_z + 2b_6\tilde{E}_\theta \tilde{E}_r, \end{aligned} \end{aligned}$$

where $b_1, \dots ,b_6$ are material parameters and $\tilde{E}_\theta$, $\tilde{E}_z$, and $\tilde{E}_r$ are the deviatoric Green–Lagrange strain components in the circumferential, axial, and radial directions, respectively. Each component $\tilde{E}_m$ $(m=r,\theta ,z)$ is defined as

18 $$\begin{aligned} \tilde{E}_m = \frac{1}{2}(\tilde{\lambda }_m^2-1), (m=r, \theta , z), \end{aligned}$$

where $\tilde{\lambda }_m$ is the deviatoric stretch ratio ($\tilde{\lambda }_m=J^{-\frac{1}{3}}\lambda _m$) (Simo and Taylor 1991).

The volumetric part of the strain energy density function, $W_\textrm{vol}$, represents the energetic resistance to macroscopic volumetric deformation of the porous skeleton associated with changes in porosity. Accordingly, $W_\text {vol}$ is defined as

19 $$\begin{aligned} \begin{aligned} W_\text {vol}&= {\left\{ \begin{array}{ll} K_v\left( \exp \left( f(J)\right) -1\right) & \text {if } J \ge 1, \\ \frac{K_v}{2}\left( \frac{J^2-1}{2}-\ln {J}\right) & \text {if } J < 1, \end{array}\right. }\\ f(J)&={}\beta (J-1)^\gamma +\frac{(J-1)^2}{2}, \end{aligned} \end{aligned}$$

where $K_v$, $\beta$, and $\gamma$ are material parameters. The piecewise definition of $W_\text {vol}$ is adopted to prevent nonphysical volumetric deformation under compression while allowing nonlinear volumetric expansion in response to transmural pressure, as suggested by recent experimental observations. The present form was selected to ensure constitutive consistency, following (Doll and Schweizerhof 2000). A comparison with a conventional volumetric penalty formulation is provided in Supplementary Material 1.

The effective Cauchy stress components ($\sigma {\prime}_m, m=r, \theta$) are obtained from the strain energy density function *W* as

20 $$\sigma _m^{\prime } = \frac{\lambda _m}{J}\frac{\partial W}{\partial \lambda _m}.$$

#### Constitutive law: interstitial fluid

The permeability of the porous solid skeleton *k* is assumed to depend on the fluid volume fraction $\phi _\text {f}$. Here, the Kozeny–Carman equation summarized in Auton and MacMinn (2017) is adopted, given by

21 $$\begin{aligned} k = k_0\frac{(1-\phi _{\text {f},0})^2}{\phi _{\text {f},0}^3}\frac{\phi _\text {f}^3}{(1-\phi _\text {f})^2}, \end{aligned}$$

where $k_0$ denotes the hydraulic permeability in the reference configuration.

### Computation

For finite element computations, the weak form of the mechanical balance equation (eq. 15) is derived as

22 $$ \int _a^b{\left( \sigma {\prime}_r r\frac{\partial w}{\partial r}+w\sigma {\prime}_\theta -\frac{Q w}{2\pi k}\right) dr} = 0, $$

where *w* denotes the weight function. The effective traction on the inner and outer surfaces is set to zero since the solid skeleton is assumed to be free from external mechanical constraints at the boundaries. The transmural pressure difference $\Delta p$ is prescribed, and the interstitial fluid flow rate *Q* is determined from eqs. 8 and 14, given by

23 $$\begin{aligned} \Delta p = \frac{Q}{2\pi }\int _a^b{\frac{1}{kr}dr}. \end{aligned}$$

In addition, the current inner and outer radii *a* and *b* must satisfy the following geometric constraints:

24 $$\begin{aligned}&u_\text {s}(a) = a - a_0, \end{aligned}$$

25 $$\begin{aligned}&u_\text {s}(b) = b - b_0. \end{aligned}$$

The computational domain [*a*, *b*] was discretized into *N* two-node line elements of equal length. The weak form (eq. 22), together with the constraint conditions (eqs. 23–25), was discretized using the Galerkin finite element method and solved for $u_\text {s}$, *Q*, *a*, and *b* under a prescribed $\Delta p$. The resulting nonlinear system was solved using Newton’s method, where the tangent Jacobian was evaluated by automatic differentiation (Leal 2018) at each iteration. Further details of the formulation are shown in Appendix A.

All computations were performed using an in-house C++ code, which is publicly available, as noted in the Supplementary Information, to ensure reproducibility. Code verification was performed by comparison with numerical solutions obtained using the pseudo-spectral method in Auton and MacMinn (2017), as shown in Appendix B.

### Computational conditions

#### Baseline computational settings

Mechanical responses of the arterial wall were examined under increasing transmural pressure difference $\Delta p$ ranging from 0 to 160 mmHg following the protocol reported in Sugita et al. (2020). Material parameters that were kept constant throughout the simulations are summarized in Table 1. The total number of elements *N* was set to 100.

**Table 1:** Summary of material parameters used for computation

Parameters	Values	Refs
$a_0$ [$\textrm{mm}$]	0.31	Kailash et al. (2025b)
$b_0$ [$\textrm{mm}$]	0.42	Kailash et al. (2025b)
$k_0$ [$\textrm{mm}^4/(\textrm{N}\cdot \textrm{s})$]	2.8 $\times 10^{-3}$	Kailash et al. (2025b)
$\phi _{\text {f},0}$ [-]	0.40	Chooi et al. (2016)
$b_1$ [-]	0.9925	Chuong and Fung (1983)
$b_2$ [-]	0.4180	Chuong and Fung (1983)
$b_3$ [-]	0.0089	Chuong and Fung (1983)
$b_4$ [-]	0.0749	Chuong and Fung (1983)
$b_5$ [-]	0.0295	Chuong and Fung (1983)
$b_6$ [-]	0.0193	Chuong and Fung (1983)

#### Parameter exploration and identification

Several material parameters in the constitutive law of the solid skeleton ($C, K_v, \beta , \gamma$) cannot be determined from existing studies based on single-phase material assumptions. Therefore, a systematic sensitivity analysis was performed within the biphasic framework using the no-pre-stretch configuration as a reference case. Subsequent analyses with pre-stretch were carried out to isolate the effects of pre-stretch under the same constitutive setting.

The parameters were varied as $C\in [20,300]$ kPa, $K_v\in [5.0,50]$ kPa, $\beta \in [0.10,3.0]$, and $\gamma \in [2.0,10]$, with sampling intervals of 10 kPa, 1.0 kPa, 0.10, and 0.20, respectively (a total of 1,640,820 computational cases). For each parameter set, the apparent radial stretch $\Lambda _r$, circumferential stretch $\Lambda _\theta$ (evaluated at the middle of the artery), and volume change ratio $\mathcal {J}$ were computed as

26 $$\begin{aligned} \Lambda _r = \frac{b-a}{\bar{b}-\bar{a}}, \, \Lambda _\theta = \frac{b+a}{\bar{b}+\bar{a}}, \, \mathcal {J} = \frac{b^2-a^2}{\bar{b}^2-\bar{a}^2}, \end{aligned}$$

and compared with experimental measurements of Sugita et al. (2020). Here, the overbar denotes reference values, which were taken as those at $\Delta p$ = 15 mmHg following (Sugita et al. 2020). In the corresponding experiments (Sugita et al. 2020), $\Lambda _r$, $\Lambda _\theta$, and $\mathcal {J}$ were obtained from local measurements within the arterial wall and subsequently averaged over multiple regions and specimens. The present definitions of the apparent deformation measures are therefore intended to capture the same overall deformation trends, rather than to reproduce the exact local measurement procedure. This approximation was adopted to reduce the influence of potential biases and uncertainties associated with local measurement procedures.

To quantify the discrepancy between simulations and experiments, an L1-type relative error measure *E*(*x*) was defined as

27 $$\begin{aligned} E(x) = \frac{1}{N_p}\sum _{j=1}^{N_p}{\left| \frac{x_\text {sim}(\Delta p_j)-x_\text {exp}(\Delta p_j)}{x_\text {exp}(\Delta p_j)}\right| }, \end{aligned}$$

where $\Delta p_j$ denotes the *j*-th prescribed transmural pressure difference ($\Delta p_j$ = 40, 80, 120, and 160 mmHg), and $N_p$ is the total number of pressure levels. The parameter set that minimized the combined error $E_\textrm{tot} = E(\Lambda _r) + E(\Lambda _\theta ) + E(\mathcal {J})$ was selected as the best-fit case for the subsequent analyses.

#### Analysis of pre-stretch effects

Furthermore, the effects of circumferential pre-stretch were examined by varying $\lambda _{\text {p},\theta }$. For simplicity, the pre-stretch distribution was assumed to vary linearly along the radial direction, with its reference value defined at the mid-wall position. This simplified parametrization was adopted to systematically assess the effects of the pre-stretch distribution on the mechanical responses rather than to identify a unique residual stress profile. The gradient of $\lambda _{\text {p},\theta }$ was varied over a range from 0 to 0.15 with an interval of $1\times 10^{-4}$, and the resulting radial variation of the circumferential stretch was evaluated. To examine the effects of radial-circumferential coupling, two limiting cases of radial pre-stretch $\lambda _{\text {p},r}$ were considered ($\lambda _{\text {p},r}=1$ (case 1) and $\lambda _{\text {p},r}=1/\lambda _{\text {p},\theta }$ (case 2)), corresponding to weak and strong transverse coupling, respectively. In each case, a representative pre-stretch distribution that minimized the radial variance of $\sigma ^{\prime }_\theta$, given by

28 $$E_\text {var}= |\sigma ^{\prime }_{\theta ,\text {max}}-\sigma^{\prime }_{\theta ,\text {min}}| $$

was selected for subsequent analyses because it yielded a nearly uniform circumferential stress in the radial direction.

## Results

### Mechanical responses without pre-stretch

First, material parameter sensitivities (*C*, $K_v$, $\beta$, and $\gamma$) were evaluated based on the error measures $E(\Lambda _r)$, $E(\Lambda _\theta )$, and $E(\mathcal {J})$. Among all tested parameter combinations, the lowest-error 1% of cases ranked by the total error $E_\text {tot}$ were extracted and summarized in Table 2, together with the single best-fit case exhibiting the minimum $E_\text {tot}$. Across the entire parameter space, the errors in $\Lambda _r$, $\Lambda _\theta$, and $\mathcal {J}$ varied over a wide range. In contrast, within this lowest-error 1% group, these errors were consistently limited to within 5.66% across all cases, despite the remaining variability in individual material parameters. In the best-fit case, the combined error $E_\text {tot}$ was reduced to 4.60%, corresponding to individual errors on the order of 1–2% for each deformation measure.

**Fig. 2:**
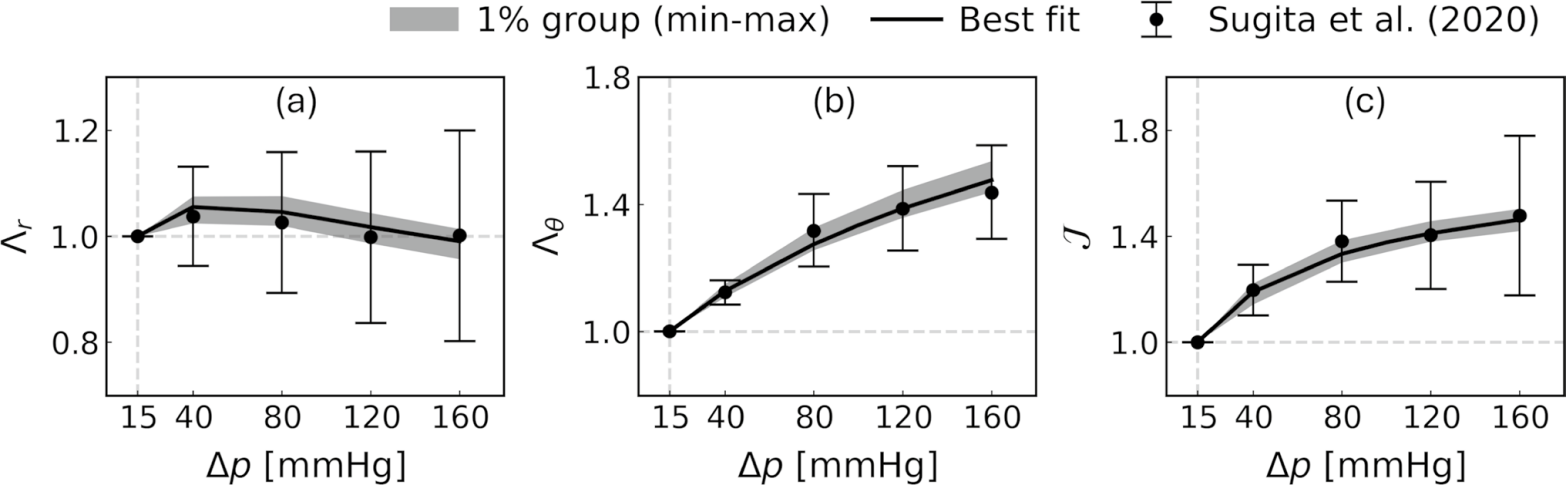
Apparent radial stretch $\Lambda _r$ **a**, circumferential stretch $\Lambda _\theta$ **b**, and the volume change ratio $\mathcal {J}$ **c** as functions of transmural pressure $\Delta p$ in the best-fit case (black lines) and the range of lowest 1% group of $E_\text {tot}$ (gray zones). Solid lines indicate simulation results, while filled circles represent experimental mean values with standard deviations reported in Sugita et al. (2020)

Figure 2 shows the apparent radial and circumferential stretches $\Lambda _r$ and $\Lambda _\theta$, together with the volume change ratio $\mathcal {J}$, as functions of the transmural pressure $\Delta p$ for the best-fit case and across the lowest-error 1% group ranked by $E_\text {tot}$. The simulation results closely matched the experimental measurements in Sugita et al. (2020) across the entire pressure range. In particular, the best-fit case showed that $\Lambda _r$ increased in the low-$\Delta p$ range up to approximately 40 mmHg, and gradually decreased with further increases in $\Delta p$, consistent with the experimental observations reported in Sugita et al. (2020).

**Table 2:** Sensitivity of material parameters to the error measures ($E(\Lambda _r)$, $E(\Lambda _\theta )$, and $E(\mathcal {J})$). Ranges are shown for all tested cases, the lowest 1% of cases ranked by the total error $E_\text {tot}$ (1% group), and the single best-fit parameter set (Best fit)

Parameters	Values (All)	1% group	Best fit
$C [\textrm{kPa}]$	[20,300]	[160,240]	210
$K_v [\textrm{kPa}]$	[5.0,50]	[5.0,24]	9.0
$\beta [-]$	[0.10,3.0]	[0.90,3.0]	1.5
$\gamma [-]$	[2.0,10]	[2.4,8.8]	4.8
$E(\Lambda _r) [\%]$	[0.377,22.0]	[0.675,3.33]	1.65
$E(\Lambda _\theta ) [\%]$	[1.09,24.6]	[1.31,3.42]	1.57
$E(\mathcal {J}) [\%]$	[0.764,29.5]	[0.798,3.22]	1.39
$E_\text {tot} [\%]$	[4.60,59.8]	[4.60,5.66]	4.60

**Fig. 3:**
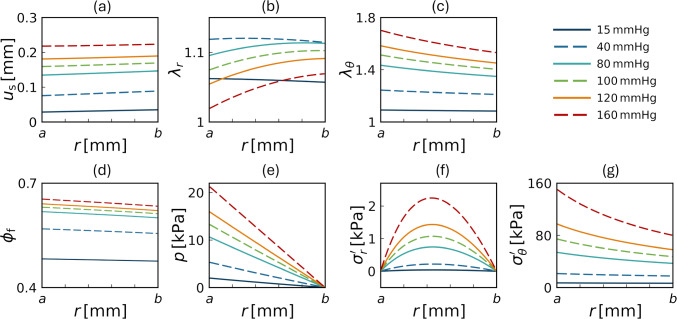
Radial distributions of $u_\text {s}$ **a**, $\lambda _r$ **b**, $\lambda _\theta$ **c**, fluid volume fraction $\phi _\text {f}$ (**d**), pore fluid pressure *p* (**e**), radial effective stress $\sigma^{\prime }_r$ (**f**), and circumferential effective stress $\sigma^{\prime }_\theta$ (**g**) at $\Delta p$ of 15, 40, 80, 100, 120, and 160 mmHg in the best-fit case

Figure 3 summarizes the radial distributions of the kinematic variables and effective stresses at different $\Delta p$ in the best-fit case, highlighting the non-monotonic radial deformation and the emergence of radial tensile stress. The radial displacement $u_\text {s}$ increased approximately linearly from the inner to the outer radius (Fig. 3a) and its magnitude monotonically increased with increasing $\Delta p$. The radial stretch $\lambda _r$ remained greater than one, indicating tensile deformation throughout the wall thickness for all pressure levels (Fig. 3b). Its magnitude increased as $\Delta p$ rose from 15 to 40 mmHg and decreased thereafter, while remaining larger than one even at higher pressures (Fig. 3b). This behavior contrasts with the circumferential stretch $\lambda _\theta$, which increased monotonically with pressure and attained larger values near the inner wall (Fig. 3c). These stretching behaviors indicate volumetric expansion of the solid skeleton and an increase in the volume fraction of the interstitial fluid $\phi _\text {f}$ (Fig. 3d).

Regarding the internal mechanical fields, pore fluid pressure *p* monotonically decreased from the inner to the outer radius and became zero at the outer surface because the outer fluid pressure was taken as the reference (Fig. 3e). Consistent with the tensile radial deformation, the radial effective stress $\sigma^{\prime }_r$ became positive at pressures above 40 mmHg and increased monotonically (Fig. 3f). These values were zero at the inner and outer radii due to the traction-free boundary conditions, and exhibited a peak near the mid-wall region. The circumferential effective stress $\sigma^{\prime }_\theta$ was approximately two orders of magnitude larger than $\sigma^{\prime }_r$ and was concentrated toward the inner wall (Fig. 3g).

### Mechanical response with pre-stretch

Next, the effects of pre-stretch on the mechanical responses were examined in the context of biphasic modeling. Here, the material parameters were taken to be the same as those of the best-fit case in Sect. 3.1.

**Fig. 4:**
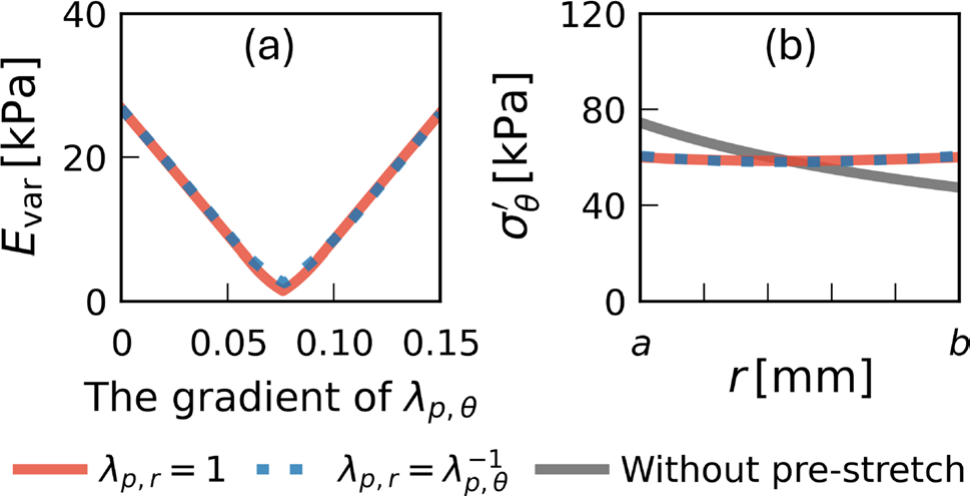
Selection of circumferential pre-stretch distributions leading to nearly uniform circumferential stress. **a** Circumferential stress variation measure $E_\text {var}(\sigma^{\prime }_{\theta })$ as a function of the radial gradient of circumferential pre-stretch $\lambda _{\text {p},\theta }$. **b** Resulting radial distributions of $\sigma {\prime}_\theta$ at the selected minima. The red solid and blue dotted lines show case 1 $(\lambda _{\text {p},r}=1)$ and case 2 $(\lambda _{\text {p},r}=1/\lambda _{\text {p},\theta })$, respectively; the gray solid line indicates the case without pre-stretch

First, a representative pre-stretch distribution was selected by minimizing the circumferential stress variation $E_\text {var}(\sigma^{\prime }_\theta )$. Figure 4a shows $E_\text {var}(\sigma^{\prime }_\theta )$ as a function of the radial gradient of the circumferential pre-stretch $\lambda _{\text {p},\theta }$ for two cases: $\lambda _{\text {p},r}=1$ (case 1) and $\lambda _{\text {p},r}=1/\lambda _{\text {p},\theta }$ (case 2). In both cases, $E_\text {var}(\sigma^{\prime }_\theta )$ exhibited local minima of approximately 2.0 kPa, and the corresponding $\sigma^{\prime }_\theta$ distribution was almost constant in the radial direction (Fig. 4b). Thus, these local minima, corresponding to nearly uniform circumferential stress distributions, were adopted for the subsequent analyses. It should be noted that the apparent stretches ($\Lambda _r$ and $\Lambda _\theta$) and volume change ratio ($\mathcal {J}$) showed results similar to those in the case without pre-stretch (Fig. 2), with total errors $E_\text {tot}$ of 4.71% (case 1) and 4.75% (case 2), respectively. Accordingly, since the inclusion of pre-stretch did not substantially affect these macroscopic deformation measures, the same material parameters identified in Sect. 3.1 were used for both cases.

**Fig. 5:**
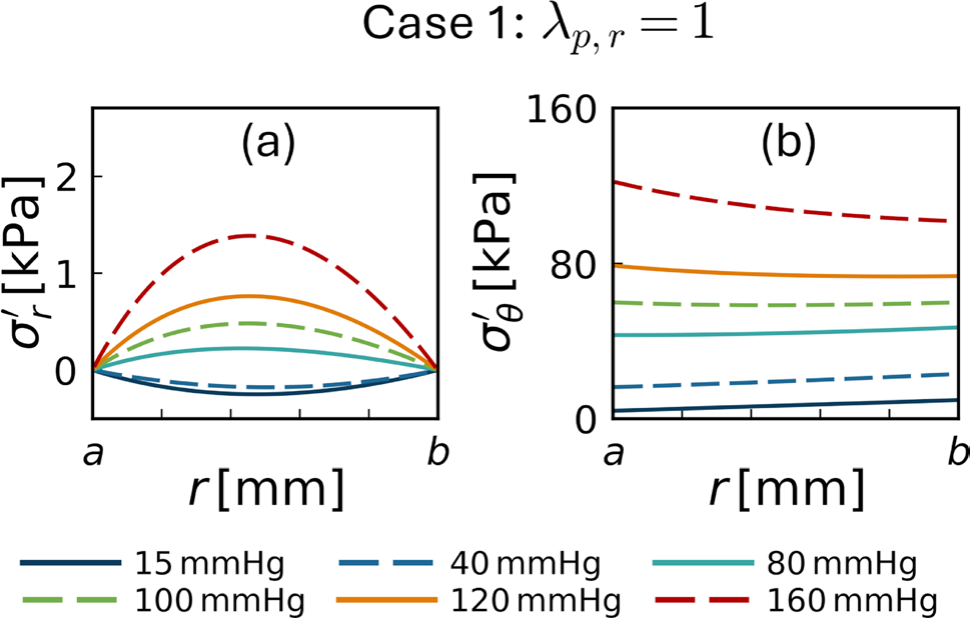
Radial distributions of the radial and circumferential effective stresses $\sigma ^{\prime }_r$ (**a**) and $\sigma ^{\prime }_\theta$ (**b**), corresponding to Fig. 3f, g but with the circumferential pre-stretch applied ($\lambda _{\text {p},r}=1$, case 1)

**Fig. 6:**
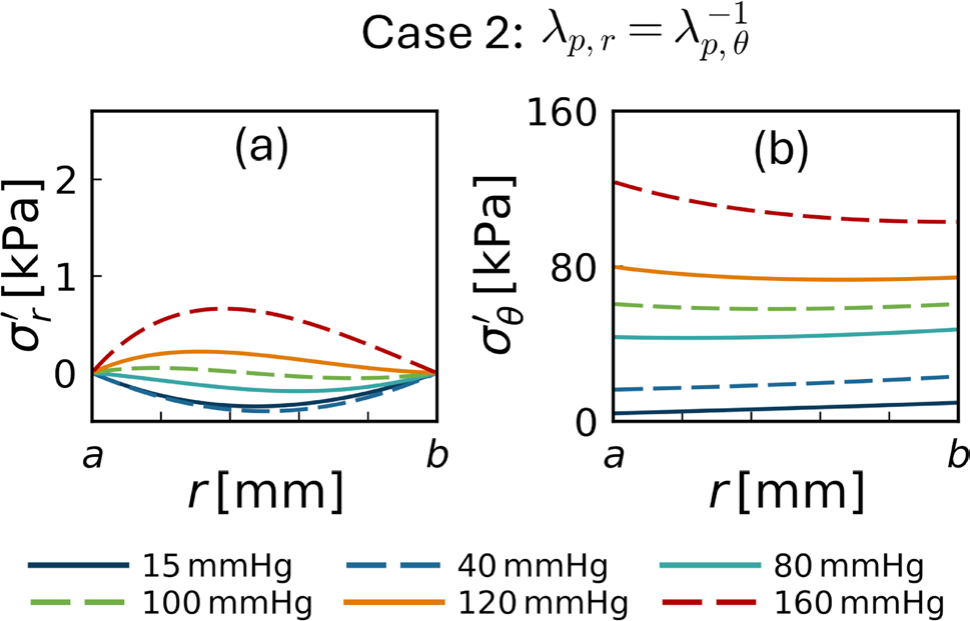
Radial distributions of the radial and circumferential effective stresses $\sigma ^{\prime }_r$ (**a**) and $\sigma ^{\prime }_\theta$ (**b**), corresponding to Fig. 3f, g but with the circumferential pre-stretch applied ($\lambda _{\text {p},r}=1/\lambda _{\text {p},\theta }$, case 2)

Figures 5 and 6 illustrate the radial distributions of the effective stresses for cases 1 and 2 with circumferential pre-stretch, respectively. In case 1 ($\lambda _{\text {p},r}=1$), the radial stress $\sigma^{\prime }_r$ was negative (compressive) at low pressures and became positive (tensile) at pressures of 80 mmHg or higher, increasing monotonically with further pressure elevation. In contrast, in case 2 ($\lambda _{\text {p},r}=1/\lambda _{\text {p},\theta }$), $\sigma^{\prime }_r$ remained negative up to approximately 100 mmHg, approached zero near this pressure level, and increased thereafter, with consistently lower magnitudes across the entire wall thickness than in case 1. The circumferential stress $\sigma '_\theta$ exhibited similar spatial profiles in both cases. Compared with the configuration without pre-stretch (Fig. 3g), the magnitude of $\sigma '_\theta$ was reduced and the radial gradient was relatively mild, even at the highest pressure level considered.

## Discussion

This study developed a biphasic modeling framework to interpret experimentally observed arterial compressibility. The constitutive law of the arterial solid skeleton incorporated the well-established radial–circumferential anisotropy (eq. 16). Although both the solid and fluid phases were assumed to be intrinsically incompressible, volumetric deformation of the solid skeleton induced by fluid–solid interactions was allowed through nonlinear volumetric behavior (eq. 19). Parameter sensitivity analyses showed that the apparent radial and circumferential stretches, as well as the resulting volume change ratios, fell within the range of experimental measurements for a subset of parameter combinations with low total errors (Fig. 2 and Table 2). These results indicate the robustness of the biphasic modeling framework in describing experimentally observed arterial deformation, including pronounced compressibility at low transmural pressures and the non-monotonic behavior of the radial stretch, beyond the scope of single-phase incompressible solid models.

In addition to reproducing arterial deformation kinematics, the biphasic modeling framework provides mechanical insights into arterial wall responses. In traditional thick-walled cylinder theory, the radial (total) stress $\sigma _r$ remains compressive to balance the applied transmural pressure. In contrast, the present biphasic framework indicates that increases in transmural pressure induce volumetric expansion of the solid skeleton (radial stretch $\lambda _r> 1$) through fluid-solid interactions. This expansion leads to the emergence of tensile radial effective stress $\sigma ^{\prime }_r$ within the solid skeleton (Fig. 3f). Although residual stress reduced the radial stress within the physiological transmural pressure range and is known to homogenize circumferential stress under physiological pressure levels (Takamizawa and Hayashi 1987; Humphrey 2002), tensile radial stress appeared at relatively high pressure levels (Figs. 5 and 6). These findings suggest a potential mechanism that may complement existing interpretations of arterial wall mechanics and pathophysiology. For instance, an intramural hematoma caused by tearing of the inner layer of the arterial wall is one of the key possibilities in the initiation of aortic dissection (Tsai et al. 2009), while conventional single-phase models, in which radial stress is always compressive, have difficulty explaining tensile loading in the radial direction, particularly at the early stage of dissection. The present biphasic framework suggests that radial tensile stress may arise under elevated transmural pressure associated with hypertension, which is a well-known risk factor for aortic dissection (Hagan et al. 2000). Thus, the present biphasic modeling framework provides a theoretical basis for exploring how fluid–solid interactions may influence mechanical responses of the arterial wall, while its direct pathological interpretation remains beyond the scope of the present simplified model.

Importantly, the present biphasic modeling framework does not contradict the conventional understanding of arterial wall mechanics but rather extends it by incorporating recently observed experimental evidence of arterial compressibility. Notably, the study by Chuong and Fung (1984), which is often cited to justify arterial incompressibility, reported volumetric changes of approximately 1–2%. Likewise, Yosibash et al. (2014) observed volumetric changes of 2–6% within the physiological pressure range. This evidence indicates that arterial tissue is not perfectly incompressible but can be reasonably approximated as nearly incompressible within the physiological pressure range. In contrast, substantially larger volumetric changes on the order of 10% have been reported under low-pressure conditions (Sugita et al. 2020) or low external loading (Nolan and McGarry 2016). To interpret these observations, biphasic modeling was applied based on well-established interstitial fluid transport mechanisms in the arterial wall. Because the arterial solid skeleton is composed primarily of collagenous fibrous tissues and an extracellular matrix rich in water, its intrinsic material behavior is expected to be nearly incompressible. Consequently, although a compressible single-phase formulation may reproduce the apparent deformation trend to some extent, it remains a more phenomenological description of the arterial wall and does not directly represent the fluid-solid interaction mechanisms within the tissue (Supplementary Material 2). Therefore, the role of the biphasic framework in the present study is not merely to improve the fit to pressure–deformation data, but to provide a physically interpretable decomposition of pore pressure, porous-skeleton deformation, and effective stress. Since biphasic modeling enables the representation of apparent arterial compressibility while preserving the intrinsic incompressibility of the solid tissue, this framework offers a reasonable approach for describing arterial biomechanics over a wide range of mechanical conditions.

It should be noted that biphasic and porous-media frameworks have been well established and extensively developed in biomechanical tissue modeling (Mow et al. 1984; Simon et al. 1993; Levenston et al. 1998; Ehlers and Markert 2001; Ehlers et al. 2009; Karajan 2009; Ateshian 2009; Tully and Ventikos 2011), including formulations that allow macroscopic volumetric deformation of the porous skeleton. These studies provide a general theoretical basis for modeling fluid-saturated soft biological tissues beyond the incompressible single-phase assumption. The present study specifically focuses on arterial wall compressibility under steady pressurization and demonstrates that a biphasic description can not only account for the experimentally observed deformation trends but also provide mechanistic insight into the internal stress states arising from fluid-solid interactions. In this sense, the novelty of the present work lies not in introducing the general concept of porous-media theory itself, but in clarifying its mechanical implications for the interpretation of arterial compressibility.

This study has four main limitations. First, the present framework was developed to interpret in vitro experiments under steady-state conditions (Sugita et al. 2020) and therefore does not account for time-dependent poroelastic effects that may arise under pulsatile arterial loading. Transient interstitial fluid transport can give rise to viscoelastic tissue responses, and further experimental evidence quantifying fluid–solid interactions is required to investigate transient mechanical behavior of the arterial wall in vivo. Second, the arterial wall was modeled as a one-dimensional system under the assumption of axisymmetric deformation, which precludes explicit representation of three-dimensional anisotropic properties determined by fiber orientations. Although the material anisotropy may not alter the qualitative behavior observed in this study, extension to fully three-dimensional formulations would be necessary to capture complex deformation modes associated with heterogeneous fiber architectures and anatomical arterial geometry. Third, the arterial wall was simplified as a single-layer structure without explicitly considering its three-layer composition. While mechanical properties of individual layers have been extensively investigated (Holzapfel and Ogden 2010b), the layer-specific mechanical interactions between the solid skeleton and interstitial fluid remain poorly understood. Incorporating layer-dependent biphasic properties represents an important direction for future studies. Fourth, the parameter set selected in Sect. 3.1 should be interpreted as a representative solution rather than a unique material identification. Because the calibration was based on a single experimental configuration and limited deformation measures, multiple parameter combinations yielded similarly small errors, as shown in Table 2. This suggests that the available data may not fully constrain all model parameters. Further experimental measurements under different loading conditions would be useful for more strongly constraining the parameter values in future studies.

## Conclusions

This study developed a biphasic modeling framework for arterial deformation under steady, axisymmetric, and plane-strain conditions and investigated the associated mechanical responses. The predicted radial and circumferential stretches, as well as the resulting volume change ratios, fell within the range of experimental measurements, demonstrating the mechanical consistency of biphasic modeling for describing pressure-induced arterial deformation. Importantly, increases in transmural pressure were shown to induce volumetric expansion of the arterial wall, leading to the emergence of tensile stress not only in the circumferential direction but also in the radial direction. Such stress states highlight mechanical features that cannot be inferred from apparent deformation measures alone and require a physically interpretable representation of fluid–solid interactions. These findings provide a mechanistic basis for interpreting arterial wall mechanics in regimes where fluid–solid interactions play a significant role.

## Supplementary Information

Below is the link to the electronic supplementary material.

Supplementary file 1 (pdf 2084 KB)

## Figures and Tables

**Fig. 7:**
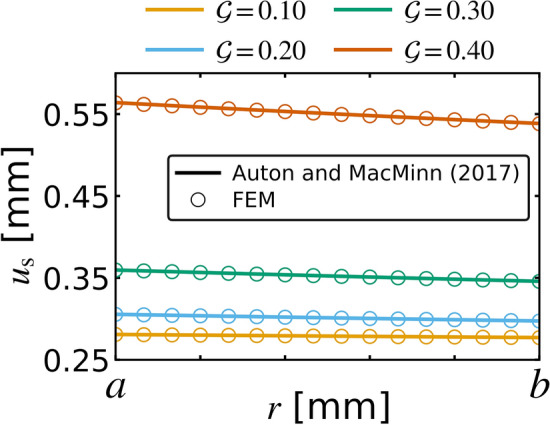
Comparison of numerical results (FEM) with semi-analytical solutions (pseudo-spectral method) in Auton and MacMinn (2017)

**Fig. 8:**
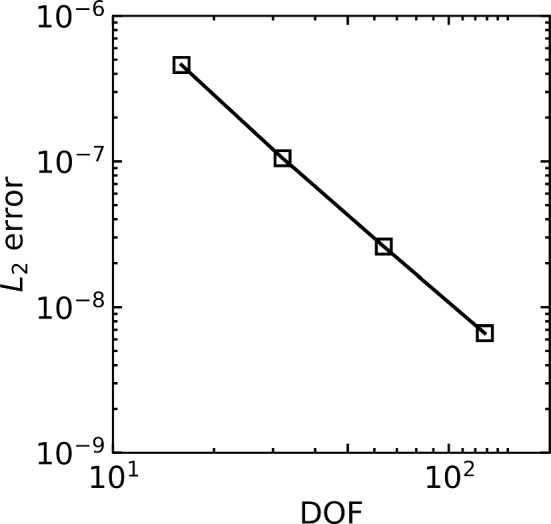
Convergence behavior of the present finite element formulation with respect to the DOF

## Data Availability

No datasets were generated or analysed during the current study. Computational code used in this study is publicly accessible from https://github.com/oubiomechlab/AxisymmetricBiphasicModeling

## Appendix A Formulation for finite element method

This section summarizes the formulation to solve eqs. 22–25 using a finite element method. Because the computational domain in eqs. 22 and 23, $r\in [a,b]$, is cumbersome to handle numerically, the coordinate was transformed to $\xi \in [0,1]$, given by

A1 $$\begin{aligned} r = a+(b-a)\xi , \frac{dr}{d\xi } = b-a. \end{aligned}$$

Using the above coordinate, the Lagrange functional $\mathcal {L}$ is defined as

A2 $$\begin{aligned} \begin{aligned}&\mathcal {L}(u_s,q,a,b,w,\psi ,\eta ,\zeta ) \\&= \int _0^1{\left( \sigma ^{\prime }_{r}r\frac{\partial w}{\partial r}+\sigma ^{\prime }_{\theta }w-\frac{qw}{k}\right) \frac{dr}{d\xi }d\xi } \\&+\psi \left( \Delta p - q\int _0^1{\frac{1}{kr}\frac{dr}{d\xi }d\xi }\right) \\&+\eta (u_s(0)-a+a_0) \\&+\zeta (u_s(1)-b+b_0), \end{aligned} \end{aligned}$$

where $\psi$, $\eta$, and $\zeta$ are Lagrange multipliers, and $q= Q/2\pi$ for simplicity. The stationary conditions with respect to *w*, $\psi$, $\eta$, and $\zeta$ are given by

A3 $$\begin{aligned}&\int _{0}^1{\left( \sigma ^{\prime }_{r}r\frac{\partial \delta w}{\partial r}+\sigma ^{\prime }_{\theta }\delta w-\frac{q\delta w}{k}\right) \frac{dr}{d\xi } d\xi }=0,\end{aligned}$$

A4 $$\begin{aligned}&\delta \psi \left( \Delta p - q\int _0^1{\frac{1}{kr}\frac{dr}{d\xi }d\xi }\right) =0,\end{aligned}$$

A5 $$\begin{aligned}&\delta \eta (u_s(0)-a+a_0) = 0,\end{aligned}$$

A6 $$\begin{aligned}&\delta \zeta (u_s(1)-b+b_0)=0, \end{aligned}$$

where $\delta$ denotes variations. Equations A3–A6 were then linearized for subsequent Newton’s method. Since Eqs. A3 and A4 become complicated because they depend on the constitutive equations for effective stress and permeability, numerical or automatic differentiation was therefore employed.

## Appendix B Code verification

The in-house code was verified by comparison with the numerical example presented in Auton and MacMinn (2017) for a nonlinear constitutive law and volume-dependent permeability without pre-stretch. The effective stress was derived from the Hencky strain, given by

B7 $$\begin{aligned} \sigma ^{\prime }_r&= \mathcal {M}\frac{\ln {\lambda _r}}{J}+\mathcal {G}\frac{\ln {\lambda _\theta }}{J} \end{aligned}$$

B8 $$\begin{aligned} \sigma ^{\prime }_\theta&= \mathcal {G}\frac{\ln {\lambda _r}}{J}+\mathcal {M}\frac{\ln {\lambda _\theta }}{J} \end{aligned}$$

where $\mathcal {M}$ and $\mathcal {G}$ are material constants. The hydraulic permeability *k* is expressed by the Kozeny–Carman equation (eq. 21).

In the computation, the computational domain was discretized with 16 two-node elements in our code, and the numerical results were compared with those obtained using the pseudo-spectral method provided in the supplementary material of Auton and MacMinn (2017), implemented in MATLAB with 200 degrees of freedom (DOFs). Convergence criteria for Newton’s iteration were set to $10^{-7}$ in both codes. Parameters used in this example were taken from Auton and MacMinn (2017), except for $\mathcal {G}$, and are summarized in Table 3. Four cases were considered with $\mathcal {G} =$ 0.1, 0.2, 0.3, and 0.4.

**Table 3:** Summary of parameters used for code verification

Parameters	Values
$a_0$ [mm]	0.85
$b_0$ [mm]	1.0
$k_0$ [$\textrm{mm}^4/(\textrm{N}\cdot \textrm{s})$]	1.0
$\phi _{\text {f},0}$ [-]	0.50
$\mathcal {M}$ [MPa]	1.0
$\Delta p$ [MPa]	0.025

Figure 7 shows the radial distribution of $u_s$ in our code and the reference solution from the pseudo-spectral method in Auton and MacMinn (2017). Both results were in excellent agreement, with a maximum relative difference below 0.04%, supporting the correctness of the present implementation.

We further examined the accuracy and mesh sensitivity of the present implementation. Figure 8 shows the L2 norm error of the solution with respect to the number of elements, relative to a numerical solution with a total of 1024 DOFs used as the reference. The error was less than $10^{-6}$ even with 16 DOFs and consistent with second-order convergence (slope = −2.03). The number of Newton iterations required for convergence was lower than 10 for all cases. These results indicate that the present FEM implementation provides numerically consistent and accurate solutions for the class of problems considered in this study, and is therefore suitable for the systematic parametric analyses. In the main analyses, the number of elements was set to 100 to obtain the smooth and continuous profiles of the variables computed within elements and the tolerance for the Newton–Raphson iteration was set to $1 \times 10^{-8}$. The computational time required for convergence was less than 1 s per case on an Intel Core i9-14900KF CPU, even without parallelization.
