# Supplementary material for: A biphasic modeling framework for arterial compressibility under steady axisymmetric deformation
Source: Biomech Model Mechanobiol. 2026 Jun 5;25(3):62. doi: 10.1007/s10237-026-02082-6 (PMC13241433; doi:10.1007/s10237-026-02082-6)
Supplement: Supplementary file 1 — (pdf 2084 KB) [file 10237_2026_2082_MOESM1_ESM.pdf]

Supplementary Material: A biphasic modeling framework for arterial compressibility under steady axisymmetric deformation

T. Fujiwara, S. Sugita, S. Wada, and T. Otani

SUPPLEMENTARY MATERIAL 1: CASES OF CONVENTIONAL VOLUMETRIC STRAIN ENERGY DENSITY FUNCTION

Additional computations to examine the sensitivity of the predicted volumetric response to the choice of W_{vol} were performed in the same manner as in the main text, except for the choice of W_{vol}. Here, we selected the following form as a representative conventional volumetric penalty function (e.g., [1]), given by

$$W_{\mathrm{vol}}(J) = \frac{K_v}{2}\left(\frac{J^2-1}{2} - \ln J\right). \tag{1}$$

Figure S1 shows the relationships between transmural pressure Δp and the apparent deformation measures Λ_r, Λ_θ, and $\mathcal{J}$ obtained using this conventional formulation for representative values of K_v, together with the experimental observations [2] and the results obtained using Eq. (19) in the main text. These results show that the conventional volumetric penalty formulation did not reproduce the experimentally observed trend of $\mathcal{J}$ as effectively as the piecewise form adopted in the main text, for the range of representative K_v values examined here. One possible reason is that this conventional energy function is primarily designed to prevent nonphysical compressive deformation ($W_{\mathrm{vol}} \to \infty$ as $J \to 0^+$), rather than to represent the nonlinear expansion behavior observed in the present experiments.

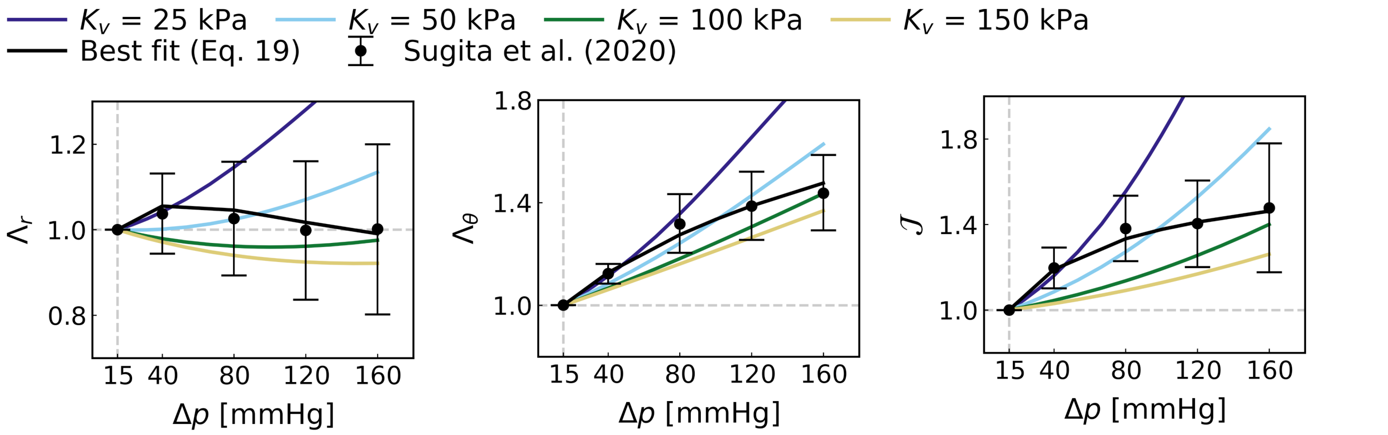

Figure S1. Relationship between transmural pressure Δp and the apparent deformation measures Λ_r, Λ_θ, and $\mathcal{J}$ obtained using the conventional volumetric penalty function for representative values of K_v, together with the experimental observations and the results obtained using Eq. (19) in the main text.

SUPPLEMENTARY MATERIAL 2: CASES OF SINGLE-PHASE SOLID MODELING

To examine the mechanical implications of the biphasic modeling framework, additional computations were performed using a compressible single-phase model under the same computational setting as in the main text. For the single-phase model, the mechanical equilibrium equation is given by

$$\frac{\partial \sigma_r}{\partial r} + \frac{\sigma_r - \sigma_\theta}{r} = 0, \tag{2}$$

where the Cauchy stress components were used directly, and the fluid pressure term in Eq. (12) of the main text was omitted. The same constitutive form of the strain-energy density function (Eqs. 16–19 in the main text) was used for the solid material, and the material parameters were tuned in the same manner to reproduce the experimental deformation measures. The transmural pressure difference was imposed through the mechanical boundary conditions for the total Cauchy stress, i.e., $\sigma_r(a) = -\Delta p$ at the inner surface and $\sigma_r(b) = 0$ at the outer surface, using the outer pressure as the reference.

As expected, the apparent deformation measures obtained by the single-phase model were also in good agreement with the experimental data (Fig. S2). However, radial compressive stress appeared due to the Poisson effect, in contrast to the tensile radial stress predicted by the biphasic model (Fig. S3). These results demonstrate that, although the apparent global deformation can be reproduced by both the compressible single-phase and biphasic models, the predicted internal mechanical fields are markedly different. Because the arterial wall is a fluid-saturated tissue, a biphasic description provides a more physically meaningful framework for examining how fluid-solid interactions influence the internal stress state. Thus, this comparison should not be interpreted as showing equivalence between the two modeling frameworks. Rather, it indicates that apparent deformation data alone are insufficient to distinguish the underlying mechanical mechanism, and that a biphasic framework is required when the objective is to interpret the role of interstitial fluid pressure and fluid-solid interaction in the internal stress state.

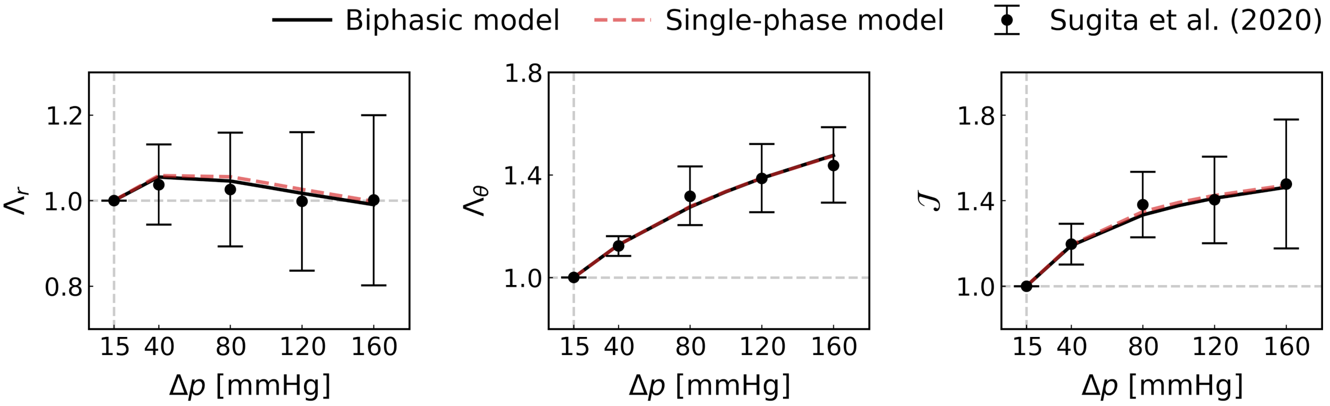

Figure S2. Apparent radial stretch Λ_r (a), circumferential stretch Λ_θ (b), and the volume change ratio $\mathcal{J}$ (c) as functions of transmural pressure Δp, corresponding to Fig. 2 in the main text. Red lines indicate the results using the compressible single-phase model.

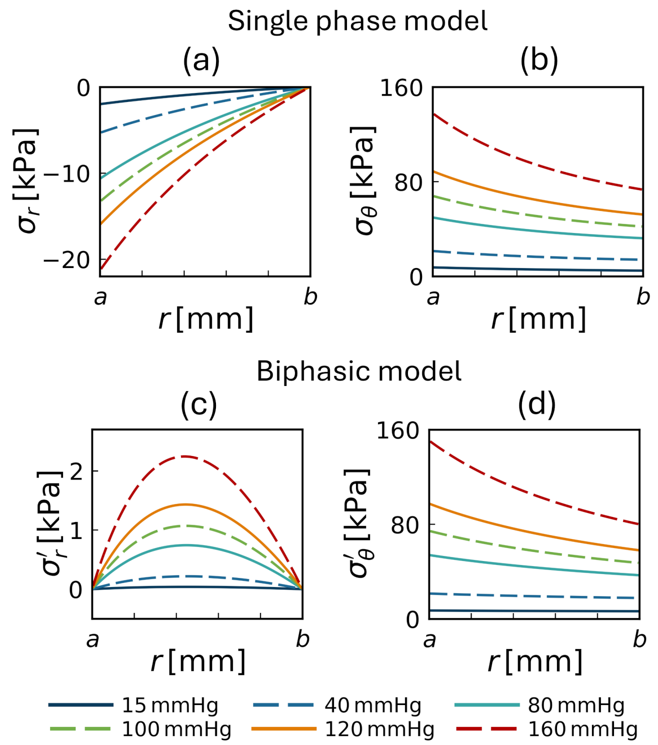

Figure S3. Radial distributions of the radial and circumferential stresses in the compressible single-phase model, σ_r (a) and σ_θ (b), and the corresponding radial and circumferential effective stresses in the biphasic model, σ'_r (c) and σ'_θ (d). The biphasic results correspond to Fig. 3f and 3g in the main text and are shown here for comparison of the internal stress states.

[1] K. A. Kailash, S. R. Akanda, A. L. Davis, C. L. Crandall, L. A. Castro, L. A. Setton, and J. E. Wagenseil, Biomechanics and modeling in mechanobiology **24**, 93 (2025).
[2] S. Sugita, M. Kato, F. Wataru, and M. Nakamura, Biomechanics and modeling in mechanobiology **19**, 147 (2020).
